# Psychosocial Stress Increases Salivary Alpha-Amylase Activity Independently from Plasma Noradrenaline Levels

**DOI:** 10.1371/journal.pone.0134561

**Published:** 2015-08-06

**Authors:** Liubov Petrakova, Bettina K. Doering, Sabine Vits, Harald Engler, Winfried Rief, Manfred Schedlowski, Jan-Sebastian Grigoleit

**Affiliations:** 1 Institute of Medical Psychology and Behavioral Immunobiology, University of Duisburg-Essen, Essen, Germany; 2 Department of Clinical Psychology & Psychotherapy, Philipps-University Marburg, Marburg, Germany; 3 Laboratory of Neuronal Structure and Function, The Salk Institute for Biological Studies, La Jolla, California, United States of America; Max Planck Institute of Psychiatry, GERMANY

## Abstract

Salivary alpha-amylase activity (sAA) and plasma noradrenaline (NA) concentrations are often considered to be surrogate markers of sympathetic activation in response to stress. However, despite accumulating evidence for a close association between sAA and noradrenaline and other indicators of sympathetic activity, reliability and generality of this relation remains unclear. We employed the Trier Social Stress Test (TSST) in order to directly compare the responses in sAA and NA to psychological stress in healthy volunteers (n = 23). The TSST significantly increased sAA and NA plasma levels with no significant differences in females and males. However, when subjects were divided according to their NA responses into low versus high responders, both groups did not significantly differ in their sAA before, during or after stress exposure. These data suggest that in response to acute psychological stress both plasma NA levels and sAA reflect sympathetic activity, however seemed to increase independently from each other.

## Introduction

Salivary alpha-amylase (sAA) is one of the major enzymes in the oral cavity. Beyond its primary function, the hydrolysis of starch and glycogen, it is involved in defense against bacteria with low sAA activity being related to a higher risk of oral infection [1]. In addition, stress-induced increases in sAA activity suggest sAA as surrogate marker of sympathetic activation [2].

Since salivary cortisol became the standard indicator of hypothalamic-pituitary-adrenal (HPA) axis activity, a comparably easy-to-use salivary measure for activity of the sympathetic-adrenal medullary system (SAMS) is highly desired. First evidence that stress-induced changes in sAA activity in humans may be dependent on beta-adrenergic transmission came from a study in which an increase in sAA activity in response to a cold water stressor was prevented by beta-adrenergic antagonists [3]. Subsequent work demonstrated changes in sAA activity induced by stress or noradrenaline infusion [2].

However, there is no „gold standard”of sympathetic nervous system (SNS) activity quantification and even the most widely accepted markers of SNS activity (heart rate and heart rate variability, blood pressure, skin conductance, plasma noradrenaline (NA) and adrenaline levels) often neither do correlate with each other, nor with sAA activity [4, 5]. In addition, some studies showed associations between sAA and NA in response to acute psychological stress [6, 7], while others failed to document such relationship [8]. Hence, even though sAA activity increases in response to stress, its potential role especially as a quantitative marker reflecting SNS activity is still lacking final evidence.

**Fig 1:**
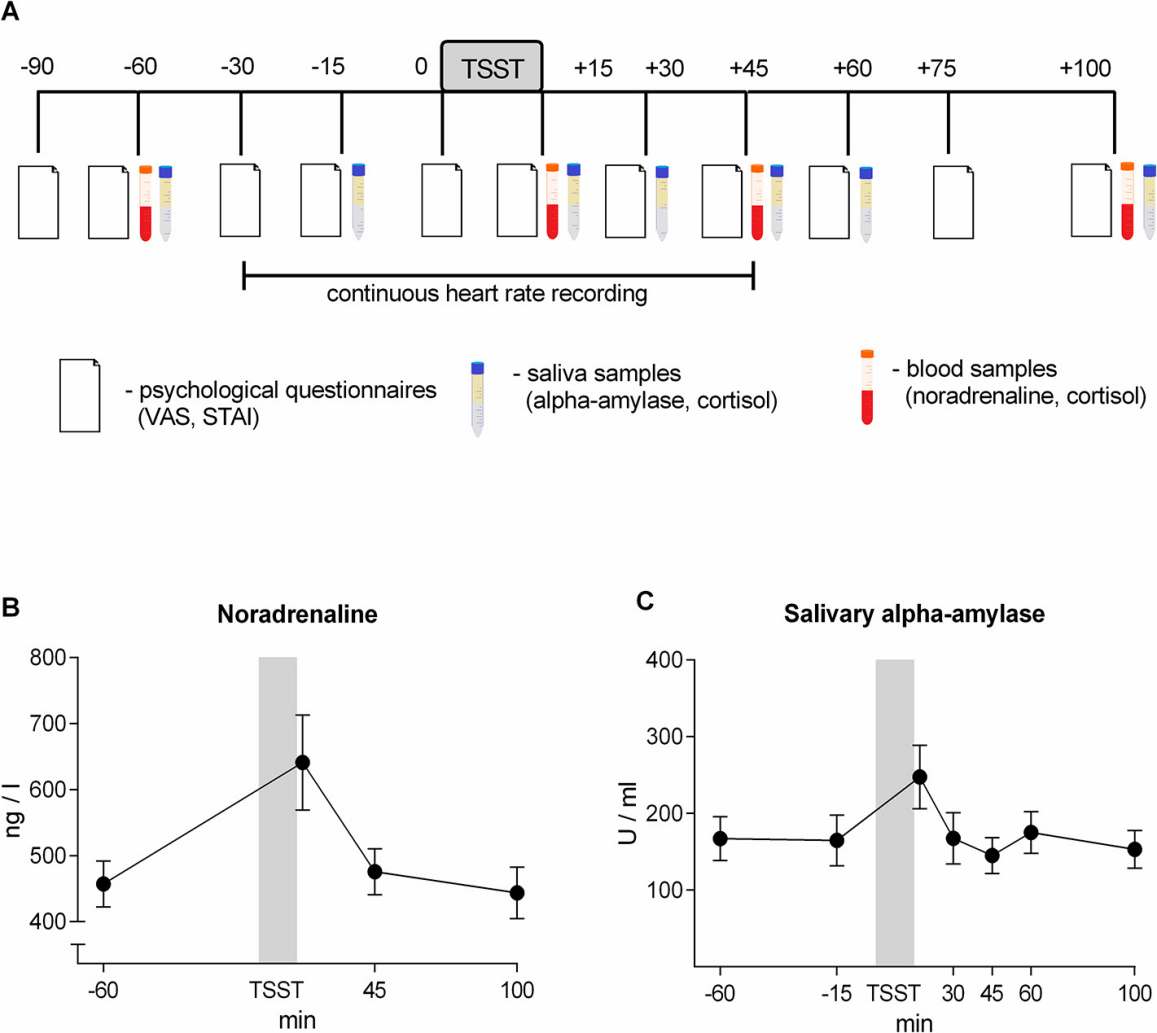
(A) Experimental design. (B) Noradrenaline and (C) salivary alpha-amylase response to the Trier Social Stress Test. Results are presented as mean ± SEM.

In this study the well-established Trier Social Stress Test (TSST) [9] was employed to induce acute psychosocial stress in healthy female and male human volunteers. Individual self-rated stress response, state anxiety, heart rate, sAA activity, salivary and plasma cortisol levels and plasma concentrations of noradrenaline (NA) were analyzed before and at various time points after the TSST (Fig 1A). Subgroups of subjects with high or low stress-induced NA responses were generated in order to analyze whether plasma NA is related to the observed increase in sAA activity.

## Materials and Methods

### Ethics Statement

The study was conducted according to Good Clinical Practice and the Declaration of Helsinki and was approved by the German Competent Authority (BfArM) and the Ethics Committee of the University of Duisburg-Essen, all participants signed informed consent forms.

### Subjects

This study is part of a larger project aiming to evaluate the efficacy of a drug in experimental stress conditions (EudraCT number: 2012-002359-40, ClinicalTrials.gov identifier: NCT01703832, see also financial statement). Here we analyze data of a control group, receiving no treatment beyond the social stress paradigm itself. From the original group consisting of 32 participants, 9 were excluded due to incomplete records (absence of blood parameter results). The final group consisted of 23 healthy volunteers (9 female and 14 male, mean age = 42.0±8.8 years, BMI = 22.6±2.3 kg/m^2^, STAI-T = 34.1±6.3). In female participants, the date of the last period was recorded (4–12 days), however not the current cycle phase. Three female subjects were using contraceptives. All participants were non-smokers and were not allowed to ingest alcohol within 24 hours before the test day or concurrent medication (except for contraception). Subjects were allowed to drink water except during 30 minutes prior to collection of saliva. A standardized snack was offered at -150 min before the TSST.

### Trier Social Stress Test

All tests and analyses were performed at the same time of the day with the TSST session starting at 02:00 p.m. Baseline measures were taken while each participant rested in an individual room. Then the investigator invited the participant to the TSST room and introduced him or her to the test settings. The subject then was asked to prepare for a notional job interview for 5 min and then confronted with a committee consisting of two authoritatively and aloofly acting investigators leading the 5 minute interview session. The participant was told that the interview was recorded and committee members would do behavioral observations and document his or her behavior. The interview was followed by a 5 minute high-demanding arithmetic task (countdown from 2043 in steps of 17). After that session each subject returned to their individual room for further assessments.

### Psychometric Measures

A visual analogue scale (VAS) was used in order to determine the subjective impression of nervousness. On a 10 cm bipolar visual scale subjects had to rate how nervous they felt ranging from 0 = “not at all” to 100 = “as nervous as possible”.

The German version of the State-Trait-Anxiety Inventory [10] used in the study differentiates between temporary/emotional state anxiety versus personality trait anxiety. The two scales with 20 items each assess anxiety as a trait (STAI-T) and anxiety as a state (STAI-S). State or momentary anxiety is characterized by tension, solicitude, nervousness, uneasiness and fear of future situations. On the contrary, trait anxiety characteristics present a stable tendency of a person and are rather stable over time. Answers are given in a 4-point rating scale ranging from 1 = “not at all” to 4 = “very true”.

### Vital Parameters and Sample Collection

For each participant, heart rate was recorded continuously, from 30 min before the test to 45 min after seated in an upright position using a Task Force Monitor (CNSystems Medizintechnik AG, Graz, Austria). Saliva samples were collected by constant chewing on a synthetic swab for 1 min using Salivettes (Sarstedt, Nümbrecht, Germany), centrifuged at 1000 x g for 2 min and stored at -80°C. An antecubital vein catheter was inserted for intermittent blood sampling 1 h prior to the TSST session. Blood samples for determination of plasma cortisol and NA levels were collected in EDTA coated collection tubes. Blood was centrifuged at 2000 x g for 10 min, and plasma was stored at -80°C.

### Laboratory Assays

Salivary and plasma cortisol levels were analyzed using a commercially available enzyme-linked immunosorbent assay (Cortisol ELISA, IBL International, Hamburg, Germany) according to the manufacturer’s instructions. Intra- and inter-assay variance was 4.8% and 5.9% respectively, the detection limit was 0.005 μg/dL. Salivary alpha-amylase activity was determined using a commercially available enzymatic assay (Salivary Alpha-Amylase Assay Kit, Salimetrics, State College, PA, USA) according to the manufacturer’s instructions. Intra- and inter-assay variance was 2.4% and 3.5% respectively, the detection limit was 3.28 U/ml.

Plasma NA was analyzed using high pressure liquid chromatography (HPLC) and an electro-chemical detector (Chromsystems GmbH, Munich, Germany, kit number 5000), according to manufacturer’s instructions.

### Statistical Analysis

We performed Shapiro-Wilk tests to assess normality of data distribution; skewed data were normalized by log-transformation and then used for statistical analysis. The parameters HR, STAI-S and pCORT were normally distributed. In contrast, Nervousness, NA, sCORT and sAA were not normally distributed and therefore log transformed. There were no outliers according to the Grubb’s test. To assess stress responsiveness we calculated individual delta scores (peak minus baseline levels). Pearson correlation coefficients (2-tailed) were used to evaluate associations between them, as well as between measured parameters at different time points. To analyze the influence of noradrenaline reactivity on other physiological measures, the whole group was divided into two subgroups by the median of their noradrenaline delta score, and ANOVA for repeated measures was applied, followed by post hoc tests with Bonferroni correction. If the assumption of sphericity was violated, Greenhouse-Geisser correction was used. Results are shown as mean ± SEM and are considered to be significant at p < 0.05. Statistical analyses were performed using PASW Statistics 20 (IBM, Chicago, IL, USA) and GraphPad Prism 6 (GraphPad Software, San Diego, CA, USA).

## Results

**Fig 2:**
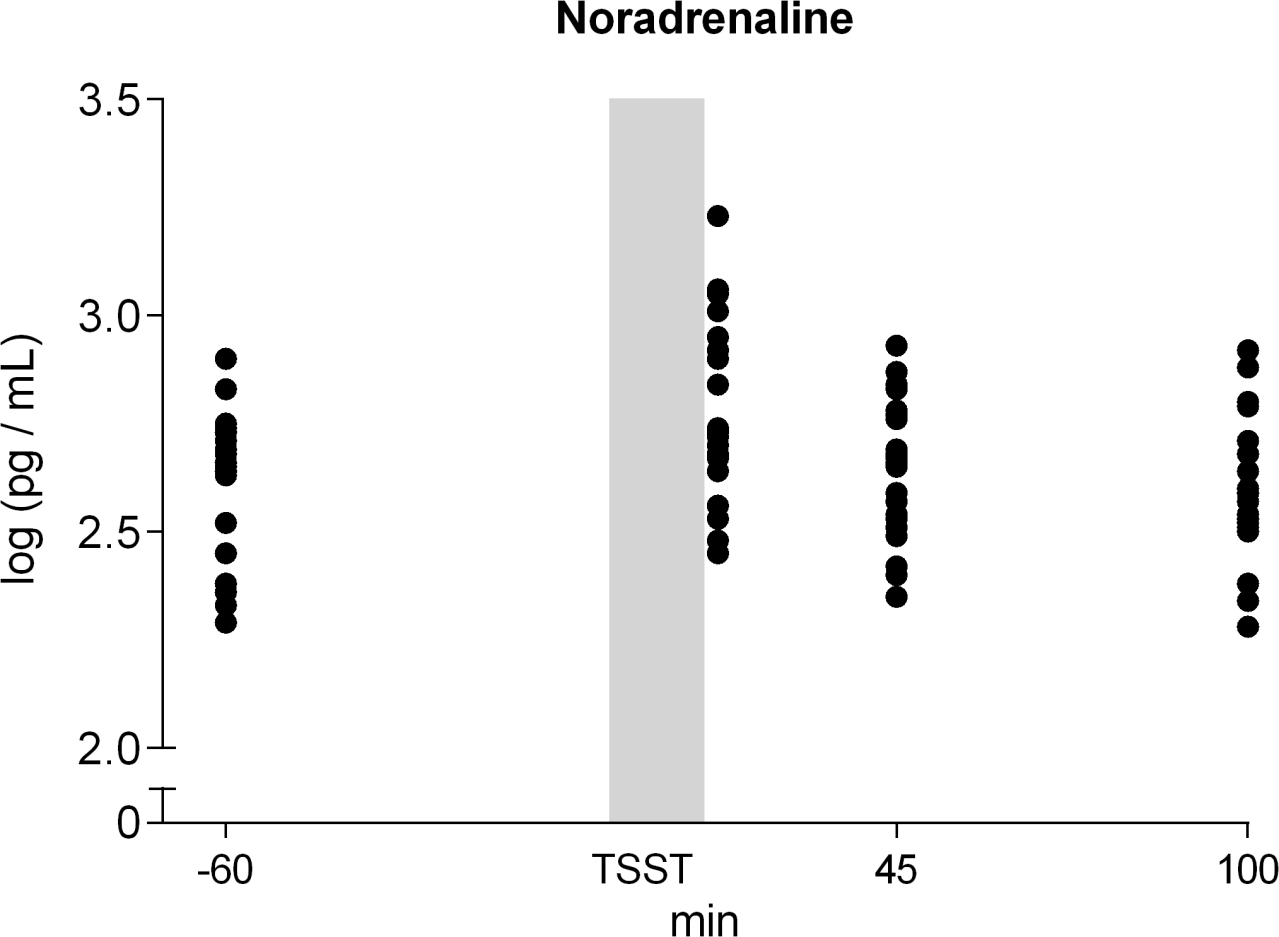
The distribution of Noradrenaline response to the Trier Social Stress Test among individuals.

The TSST caused a pronounced increase in self-reported nervousness (F (1.797, 39.543) = 30.074; p<0.001), a significant rise in heart rate (F (2.118, 46.603) = 25.127; p<0.001) and an activation of the HPA axis indicated by significantly increased levels of plasma cortisol (F (3, 66) = 15.303; p<0.001), and salivary cortisol concentrations (F (2.585, 54.275) = 14.778; p<0.001) (data not shown). Plasma NA and sAA activity peaked 15 min after the TSST and quickly returned to baseline within 30 min (F (1.612, 35.455) = 8.846; p = 0.002, and F (2.600, 57.203) = 6.190; p = 0.002 respectively; Fig 1B and 1C and 2).

To analyze the relationship between measured parameters, we calculated correlations between their level at different measurement time and between peak minus baseline level. NA did not correlate with sAA, as well as with all other parameters (p > 0.05). Nevertheless, sAA correlated with pCORT at +15 min, +45 min and +100 min time points (r = 0.585, p = 0.003; r = 0.497, p = 0.016; r = 0.514, r = 0.012, respectively). PCORT also correlated with sCORT at every measured time (-60 min: r = 0.485, p = 0.019; +15 min: r = 0.704, p < 0.001; +45 min: r = 0.737, p < 0.001; +100 min: r = 0.725, p < 0.001). Peak minus baseline correlation analysis revealed a close connection only between STAI-S and Nervousness (r = 0.53, p = 0.009).

**Fig 3:**
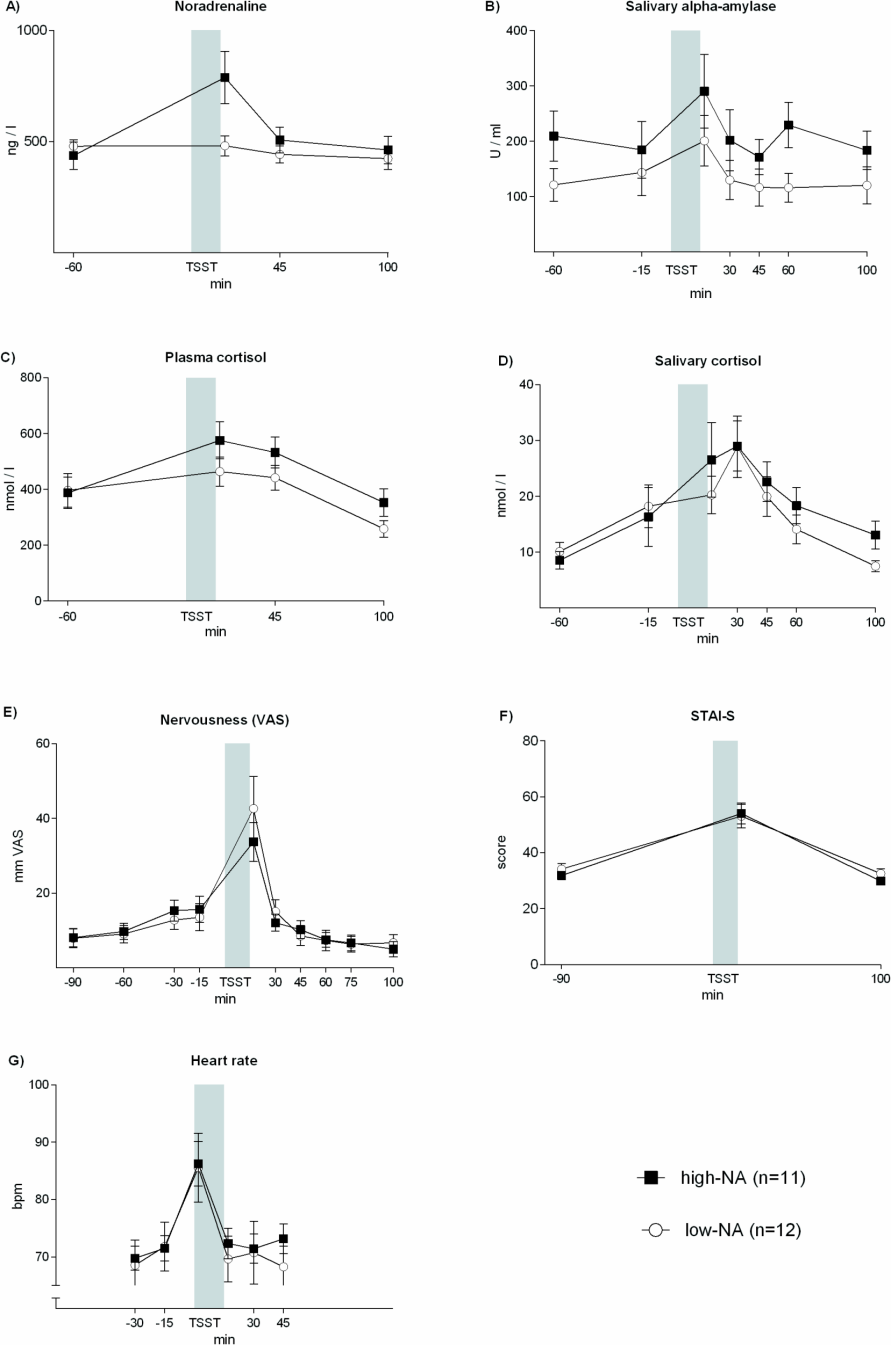
Noradrenaline (A), Salivary alpha-amylase (B), Plasma cortisol (C), Salivary cortisol (D), Nervousness (E), State anxiety (F) and Heart rate (G) in response to the Trier Social Stress Test for 2 groups with high-NA and low-NA response. Results are presented as mean ± SEM.

To determine whether and to what extent stress-induced increases in NA and sAA activity are independently reflecting stress-induced sympathetic activation, we performed a median split by NA delta scores and obtained two equally sized groups. Repeated measures ANOVA was employed to assess differences (low-NA vs high-NA) in the parameters reported. Groups significantly differed in their stress-induced noradrenaline response (high-NA vs. low-NA) (Fig 3A) (F(3, 63) = 5.516, p = 0.002; ANOVA interaction effect). Despite this distinct difference in stress-induced NA concentrations, high-NA vs. low-NA groups did not significantly differ in sAA activity level before and after the stress exposure (Fig 3B). In addition, subjects in neither group significantly differ in subjective rating of nervousness, heart rate, plasma and salivary cortisol (all p > 0.05) or other measures (p > 0.05, Fig 3C–3F). There were no differences in age, BMI, and STAI across male and female participants. Nor were there such differences between NA high- and low/non-responders.

## Discussion

SAA activity increasing in response to stress is often considered a non-invasive marker of sympathetic activation. However, after more than three decades of research addressing this issue (with a significant rise in the number of studies during the past couple of years), reliability of this marker still lacks final evidence [2, 5]. This might be due to differences in the various stress paradigms and experimental setups used as well as to the lack of a „gold standard”for sympathetic activity to validate measures of sAA [4, 11]. In this study we employed the TSST as a widely accepted and standardized paradigm for the induction of social stress to evaluate whether a rise in plasma NA levels is necessary or predictive of the elicited increase in sAA activity. We found substantial diversity in the NA responses of our subjects, allowing us to median-split the group into two subsets either containing moderate to high- or low to virtually non-responding individuals. However, subjects in both subgroups expressed comparable rises in sAA activity and sAA correlated with pCORT, but not with NA or any other parameter we assessed, which indicates a connection between HPA axis and sAA regulation.

Several studies demonstrated increases in plasma NA, sAA, and both plasma and salivary cortisol levels in response to the TSST [11]. However, of the impressive number of studies employing the TSST only a few examine sAA together with another potential SAMS marker and those studies show divergent results [2, 5, 11].

Inconsistencies between results from different studies may be due to differences between employed stressors: while psychological stressors predominantly activate the HPA axis, the SAMS is more susceptible to physiological stressors [11]. The diversity in NA response among subjects indicates a complex sympathetic response to psychological stress. It was shown previously, that mental stress increases blood pressure and heart rate while altering muscle sympathetic nerve activity in both directions or not changing it at all [12]. Despite a large number of stress studies, the mechanisms of sympathetic response are not fully understood.

Previous work from our group using endotoxin administration as a physiological stressor revealed a correlation between NA and sAA responses [13] and Chatterton at al. demonstrated a significant association between catecholamine levels and sAA in physiological but not psychological stress conditions [14]. Considering this argumentation our results are still in conflict with those from a similar study that found sAA activity to be predictive of NA levels in TSST subjects [7]. This inconsistency might be in part explained by the complex and susceptible nature of the TSST and its dependency on individual subjects and exact test settings (e.g. committee members, blood drawings etc.) or some of the following limitations: salivary glands’ output is not only regulated by the sympathetic (SNS) but also by the parasympathetic nervous system (PNS) which had been shown to decrease sAA activity after sole activation but enhance it during concomitant SNS activation [5, 15, 16]. Little is known about sympathetic-parasympathetic balance during and after the TSST, and we cannot exclude that PNS-influences masked or modified an existing relation between the sAA and noradrenaline response. Further limitations may derive from methodological problems: unlike salivary cortisol sAA is not derived from the blood but actively secreted into the saliva, which makes its concentration dependent from the salivary flow rate. Due to this it is an ongoing matter of debate which collection method provides the most accurate results [17]. In our study we used Salivettes for saliva collection with chewing of a synthetic swab for a defined time interval. Some researchers recruit subjects with the same gender, taking into consideration reports of altered psychological and physiological stress response in men and women [18]. However, we did not find gender effects on any of the measured parameters.

In conclusion, sAA activity clearly increases in response to psychosocial stress. However, in this study we did not find an association between plasma noradrenaline response and sAA. Our data suggest that the relation between sAA activity, NA release and sympathetic activity is complex and differs between different types and models of stress. Therefore reliability of sAA as a distinct or even quantitative marker of sympathetic activity remains questionable.
